# Psychological Symptoms in Patients with Inborn Errors of Immunity and Their Family Members: A Cross-Sectional Study

**DOI:** 10.3390/medicina62081616

**Published:** 2026-08-21

**Authors:** Gulistan Alpagat, Necip Nas

**Affiliations:** 1 Department of Clinical Immunology and Allergic Diseases, Malatya Training and Research Hospital, 44900 Malatya, Türkiye; 2 Department of Internal Medicine, Private Siirt Duru Hospital, 56100 Siirt, Türkiye; necipnas@gmail.com

**Keywords:** Inborn errors of immunity, primary immunodeficiency disorders, anxiety, caregiver burden, psychosocial health, Beck Anxiety Inventory, Beck Depression Inventory

## Abstract

*Background and Objectives***:** Inborn errors of immunity (IEI), also referred to as primary immunodeficiencies, are chronic conditions characterized by recurrent infections and long-term treatment requirements that may adversely affect psychological well-being. Although mental health outcomes in patients with IEI have been increasingly recognized, data regarding the psychological impact on family members remain limited. This study aimed to evaluate anxiety and depressive symptoms in patients with IEI and their family members compared with healthy controls and to examine the association of sociodemographic and clinical factors with anxiety and depressive symptom scores. *Materials and Methods***:** A total of 375 participants were included, comprising 34 patients with IEI, 102 family members, and 239 healthy controls. Anxiety and depressive symptoms were assessed using the Beck Anxiety Inventory (BAI) and the Beck Depression Inventory (BDI). Group comparisons were performed using non-parametric statistical tests. Multivariable linear regression analyses were conducted to identify independent predictors of anxiety and depressive symptoms. The internal consistency of the scales was evaluated using Cronbach’s alpha, McDonald’s omega, and Guttman’s lambda-2 coefficients. *Results***:** Both patients with IEI and their family members demonstrated significantly higher BAI and BDI scores than healthy volunteers in the unadjusted comparisons (all *p* < 0.001), with no statistically significant differences between patients and relatives. After multivariable adjustment using HC3 robust standard errors, healthy volunteers had significantly lower BAI and BDI scores than patients with IEI. Patient relatives had significantly lower BDI scores, but their adjusted BAI scores did not differ significantly from those of patients. Female sex remained independently associated only with higher BAI scores. The BAI and BDI showed excellent internal consistency, with all reliability coefficients exceeding 0.90. *Conclusions***:** Patients with IEI and their family members had higher anxiety and depressive symptom scores than healthy volunteers. Although patients and relatives had comparable BAI and BDI scores in the unadjusted comparisons, adjusted analyses showed significantly lower BDI scores among relatives, whereas the adjusted BAI difference was not statistically significant. Healthy volunteers had significantly lower adjusted BAI and BDI scores than patients, and female sex was independently associated with higher BAI scores. These findings support consideration of psychological well-being at both the patient and family levels in IEI, although the cross-sectional design precludes causal inference.

## 1. Introduction

Inborn errors of immunity (IEIs), formerly referred to as primary immunodeficiency disorders, comprise a heterogeneous group of disorders characterized by defects in immune system development or function and a broad spectrum of clinical manifestations, including recurrent or severe infections, immune dysregulation, autoimmunity, autoinflammation, allergy, and malignancy [1,2,3,4,5]. The clinical and genetic spectrum of IEIs continues to expand, as reflected in the most recent International Union of Immunological Societies (IUIS) Expert Committee classification [5]. The chronic and heterogeneous clinical course of IEIs may be accompanied by substantial psychosocial challenges for affected individuals.

Psychiatric morbidity has been increasingly recognized in IEI, with population-based evidence demonstrating an increased risk of psychiatric disorders and suicidal behavior among individuals with primary humoral immunodeficiencies [6]. The psychological vulnerability of individuals with IEI may reflect the interaction of multiple factors. Recurrent infections, diagnostic uncertainty or delay, chronic disease manifestations, and the need for long-term treatment may contribute to persistent psychological stress. In parallel, emerging evidence suggests that immune dysregulation and chronic inflammatory processes may be associated with neuropsychiatric manifestations, providing a potential biological link between altered immune function and psychological symptoms in affected individuals. These mechanisms should, however, be regarded as potential contributors rather than established causal pathways. Anxiety and depressive symptoms may therefore represent clinically relevant aspects of the broader psychosocial experience of living with IEI. Importantly, the psychological implications associated with IEI may extend beyond affected individuals, although biological mechanisms proposed for patients do not directly explain psychological symptoms among their family members.

Psychiatric and psychosocial outcomes have consequently received increasing attention in IEI-specific research. Heath et al. reported clinically relevant anxiety and depressive symptoms among adults with primary immunodeficiency and found that patients attributed a substantial proportion of these symptoms to their underlying immunodeficiency [7]. Importantly, the psychological implications of IEI may also extend beyond affected individuals. Increased anxiety and depressive symptoms among mothers of children with primary immunodeficiency, together with measurable caregiver burden [8]. Similarly, higher anxiety and depression levels and impaired aspects of health-related quality of life among parents of children with primary immunodeficiency [9]. These findings highlight the relevance of considering psychological well-being within the broader family context. However, existing family-focused studies have predominantly examined parents or caregivers of pediatric patients, leaving limited evidence regarding adult family members of adults with IEI.

Although anxiety and depressive symptoms have increasingly been recognized among adults with IEI, considerably less is known about these symptoms among their adult family members. Existing studies have predominantly focused on patients themselves or on parents and caregivers of pediatric patients, leaving limited evidence regarding adult relatives of adults with IEI. Moreover, few studies have simultaneously evaluated patients with IEI, their family members, and healthy controls within the same analytical framework. Therefore, this study aimed to compare anxiety and depressive symptom scores among adults with IEI, their family members, and healthy controls and to examine the associations of sociodemographic and clinical factors with these symptom scores.

## 2. Materials and Methods

### 2.1. Ethical Considerations

The study was conducted in accordance with the ethical principles of the Declaration of Helsinki and received approval from the Malatya Turgut Özal University Health Sciences Scientific Research Ethics Committee (approval date: 16 July 2025; reference no: E-30785963-020-315439). All participants were informed in detail about the study objectives and procedures prior to participation, and electronic informed consent was obtained.

### 2.2. Study Design and Participants

This study was designed as a cross-sectional observational survey involving patients with IEI, their family members, and healthy volunteers. The study was conducted between August and December 2025 at the Adult Immunology Outpatient Clinic of Malatya Turgut Özal University Training and Research Hospital. All consecutive adult patients (≥18 years) with a confirmed diagnosis of IEI who attended routine outpatient follow-up during the study period and met the predefined eligibility criteria were invited to participate. Eligibility was assessed by experienced clinical immunologists according to the predefined inclusion and exclusion criteria.

A total of 34 patients with IEI who agreed to participate and completed the questionnaire were included. The relative group comprised 102 adult family members who agreed to participate, were able to read and understand the questionnaire, and could complete it independently. Patients’ relatives were defined as individuals with a close familial or caregiving relationship to the patient, and eligible family members accompanying participating patients were invited to participate. The control group comprised 239 healthy volunteers recruited from the local community during the same study period through community-based announcements and personal contacts. Potential controls were screened according to the predefined eligibility criteria and completed the same questionnaire. Controls were not individually or frequency-matched to patients or their relatives with respect to sociodemographic characteristics. Information on whether healthy volunteers were healthcare personnel or relatives of healthcare personnel was not systematically collected.

**Figure 1:**
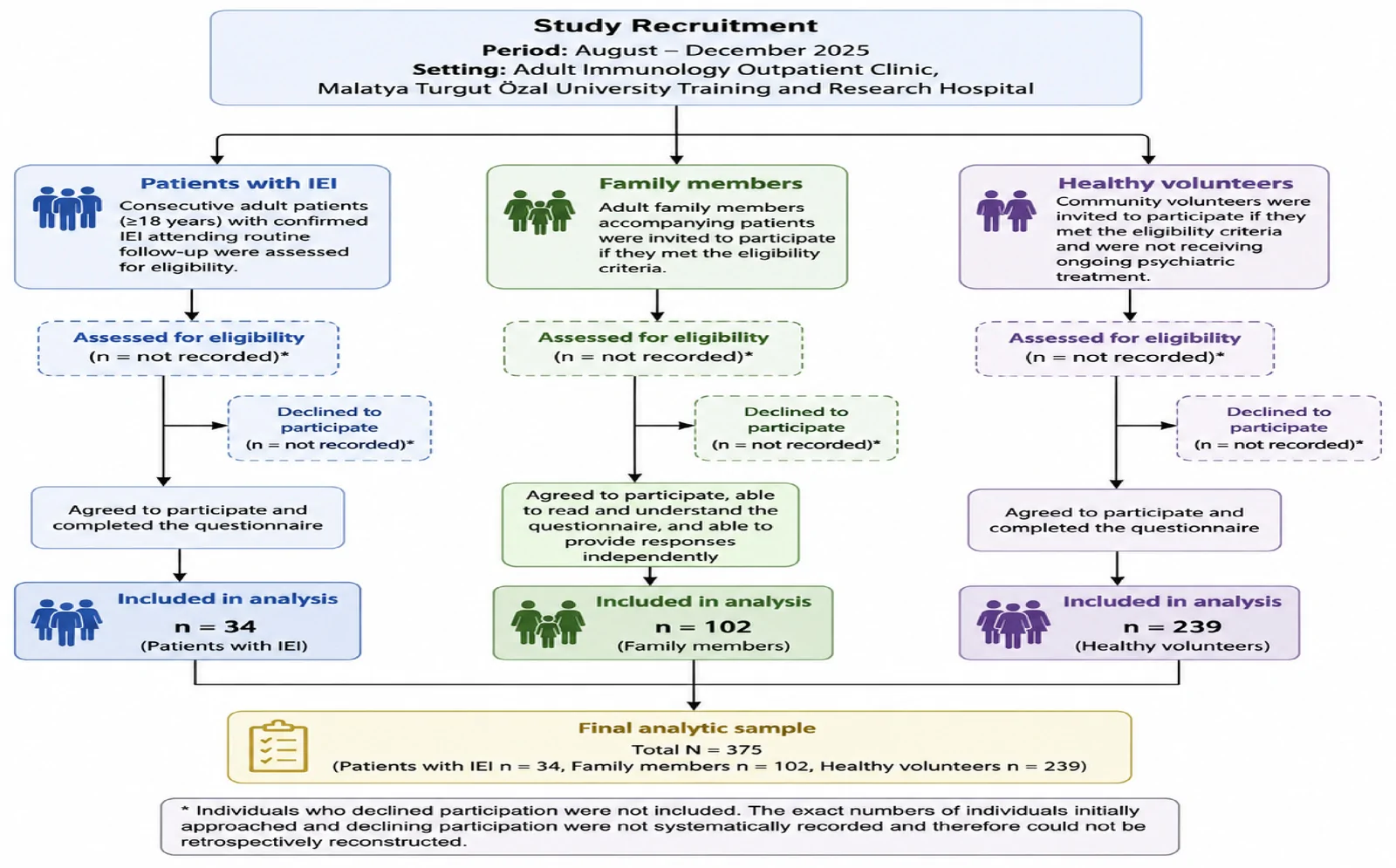
Participant recruitment and study flow. A total of 34 patients with IEI, 102 family members, and 239 healthy volunteers were included in the final analysis (*n* = 375). The exact numbers of individuals initially approached and those who declined participation were not systematically recorded and therefore could not be retrospectively reconstructed.

The same general inclusion and exclusion criteria were applied across the three study groups, except for group-specific eligibility requirements. A confirmed diagnosis of IEI was required for the patient group, whereas individuals with a known diagnosis of IEI were excluded from the control group. Active malignancy, pregnancy, and ongoing psychiatric treatment were exclusion criteria for all groups. Individuals who declined participation were not included. The exact numbers of individuals initially approached and those who declined participation were not systematically recorded and therefore could not be retrospectively reconstructed. Participant recruitment and final study-group composition are summarized in Figure 1.

Patients with IEI were clinically evaluated and classified according to the 2024 International Union of Immunological Societies (IUIS) Expert Committee update based on clinical manifestations, immunological profiles, and available molecular genetic findings [6]. Disease-related information, including IEI diagnosis and classification, age at diagnosis, follow-up duration, treatment-related characteristics, family history of IEI, and molecular genetic diagnosis when available, was obtained from clinical records and verified by experienced immunologists. For participating relatives, the relationship to the patient was recorded; however, cohabitation status, caregiving intensity, duration of caregiving, and time spent providing care were not systematically assessed.

Because the primary objective was to evaluate psychological symptoms at the overall IEI cohort level rather than to compare individual IEI diagnoses, patients with different IEI subtypes were analyzed collectively in the primary analyses. Individual diagnoses and their distribution are reported descriptively. Given the small and uneven numbers of patients across diagnostic categories, subtype-specific inferential analyses were not considered statistically reliable and were therefore not performed.

Monthly household income was self-reported and categorized according to the hunger and poverty thresholds reported by the Confederation of Turkish Trade Unions (TÜRK-İŞ) at the beginning of the recruitment period (August 2025). In August 2025, the monthly hunger threshold for a four-person household was Turkish Lira (TRY) 27,111.17 and the poverty threshold was TRY 88,309.98. For practical categorization, these values were rounded to TRY 27,000 and TRY 88,000, respectively. Accordingly, monthly household income was classified as below the hunger threshold (<TRY 27,000), between the hunger and poverty thresholds (TRY 27,000–88,000), and above the poverty threshold (>TRY 88,000) [9]. Income level was included in univariable and multivariable analyses to assess its association with anxiety and depressive symptom scores.

### 2.3. Psychological Assessment Instruments

Depressive and anxiety symptoms were assessed using validated self-report instruments: the Beck Depression Inventory (BDI) and the Beck Anxiety Inventory (BAI). The validated Turkish versions of both instruments were used. The Turkish validity and reliability of the BDI and BAI have previously been established by Hisli and by Ulusoy et al., respectively [10,11].

The BDI is a widely used instrument designed to evaluate the severity of depressive symptoms experienced over the previous week. It consists of 21 items, each scored on a 4-point Likert scale ranging from 0 to 3, yielding a total score between 0 and 63. Higher scores indicate greater severity of depressive symptomatology. The scale assesses cognitive, emotional, motivational, and somatic aspects of depressive symptoms and has demonstrated strong psychometric properties in adult populations.

The BAI was used to measure the severity of anxiety symptoms experienced during the past week, including the day of assessment. This scale comprises 21 items focusing primarily on somatic and subjective symptoms of anxiety. Each item is rated on a 4-point Likert scale (0–3), with total scores ranging from 0 to 63, where higher scores reflect increased anxiety severity. The BAI is recognized for its ability to distinguish anxiety symptoms from depressive features and has been extensively validated in clinical and non-clinical adult samples.

Both scales were administered electronically as part of the structured online questionnaire and were completed independently by participants under similar conditions. Standardized scoring procedures were applied for all respondents. The instruments were used exclusively as self-report screening questionnaires for research purposes and were not intended to establish psychiatric diagnoses.

### 2.4. Data Collection Procedure

Prior to completing the questionnaire, participants provided demographic information, including age, sex, marital status, educational level, and degree of kinship to the patient (for relatives). The questionnaire was accessed via a secure online link and completed independently by each participant. Survey responses were collected anonymously, without names, email addresses, IP addresses, or other direct personal identifiers. No participant-specific identification code was used; therefore, responses could not be linked back to individual participants. Access to the study dataset was limited to authorized members of the research team. Electronic data were stored in a password-protected account, and access was controlled through account authentication and restricted to authorized investigators only. Data collection, access, and storage procedures were conducted in accordance with the ethics-approved study protocol and applicable data-protection requirements.

#### Inclusion and Exclusion Criteria

*Inclusion Criteria*
Participants were considered eligible for inclusion if they met all the following criteria:
  • Aged 18 years or older at the time of enrollment
  • Provided voluntary informed consent to participate in the study
  • Demonstrated adequate cognitive capacity to comprehend the study objectives and to complete the questionnaire independently and reliably
*Exclusion Criteria*
Participants were excluded from the study if they met any of the following criteria:
  • Younger than 18 years of age
  • Declined or withdrew consent to participate
  • Exhibited insufficient cognitive ability to understand or accurately respond to the questionnaire
  • Were pregnant at the time of data collection
  • Had a known or previously diagnosed malignancy
  • We are receiving ongoing psychiatric treatment at the time of study enrollment.

This study was reported in accordance with the Strengthening the Reporting of Observational Studies in Epidemiology (STROBE) guidelines for cross-sectional studies.

### 2.5. Statistical Analysis

Statistical analyses were performed using JASP software (version 0.19.0; University of Amsterdam, The Netherlands). Normality of continuous variables was assessed using the Kolmogorov–Smirnov and Shapiro–Wilk tests, complemented by visual inspection of histograms and Q–Q plots. Non-normally distributed variables were analyzed using non-parametric methods. The completeness and internal consistency of the dataset were checked against the original survey records before analysis. No missing data were identified for the primary outcomes or for the variables included in the multivariable models; therefore, no imputation was performed. Kinship information was unavailable or inconsistent for two participants in the relative group. Analyses involving kinship were therefore conducted using available-case data, and the variable-specific denominator was reported. Percentages for all other variables were calculated using the total number of participants in the corresponding study group.

Continuous data are presented as mean ± standard deviation (SD) or median (interquartile range, IQR), as appropriate. Categorical variables are expressed as frequencies and percentages. Sociodemographic and clinical comparisons involving BAI and BDI scores were considered secondary exploratory analyses. To control for multiplicity across these analyses, the Benjamini–Hochberg false discovery rate procedure was applied separately to the seven sociodemographic comparisons conducted for each outcome. Both unadjusted *p* values and FDR-adjusted *p* values are reported. Where an overall comparison involving three or more categories was statistically significant after FDR adjustment, pairwise comparisons were performed using Dunn’s test with Bonferroni adjustment. Because several kinship and marital-status categories contained very small numbers of participants, these results were interpreted cautiously. Kinship categories were presented descriptively without inferential comparison.

Multiple linear regression analyses were used to evaluate factors independently associated with BAI and BDI scores. Participant group, sex, age group, chronic disease status, employment status, monthly household income, and educational level were entered simultaneously into the models based on clinical relevance and previous literature. Automated variable-selection procedures were not used. Regression assumptions were evaluated using residual-versus-fitted plots, Q–Q plots of residuals, the Breusch–Pagan test for heteroscedasticity, externally studentized residuals, leverage values, Cook’s distance, and variance inflation factors. Because the residual distribution deviated from normality and heteroscedasticity was detected in the BAI model, HC3 heteroscedasticity-consistent robust standard errors were used for both models. Unstandardized regression coefficients, robust standard errors, 95% confidence intervals, and two-sided *p* values are reported. Given the relatively small patient group and sparse categories in some covariates, the multivariable analyses were considered exploratory and the estimates were interpreted cautiously. All tests were two-tailed, and *p* < 0.05 was considered statistically significant.

## 3. Results

A total of 375 individuals, including 34 patients, 102 patient relatives, and 239 healthy volunteers, were included in the study (Table 1). BMI differed significantly among the study groups (*p* = 0.005). The groups were comparable in terms of sex distribution (*p* = 0.535), age categories (*p* = 0.148), marital status (*p* = 0.090), and smoking status (*p* = 0.070). In contrast, chronic disease prevalence differed significantly among the groups and was highest among patients (76.5%), followed by patient relatives (34.3%) and healthy volunteers (25.9%) (*p* < 0.001). Employment status also differed significantly among the groups (*p* = 0.003), with a higher proportion of healthy volunteers being employed (66.9%) compared with patient relatives (51.0%) and patients (44.1%). Monthly household income and educational level also differed significantly among the groups (both *p* < 0.001), with healthy volunteers more frequently represented in the higher-income and university-educated categories.

The clinical and treatment-related characteristics of the patient group and the kinship characteristics of participating relatives are summarized in Table 2. Of the 34 patients, 12 (35.3%) were diagnosed after 40 years of age. Immunoglobulin replacement was administered intravenously in 31 patients (91.2%), subcutaneously in two patients (5.9%), and via a facilitated subcutaneous route in one patient (2.9%). Medication-related side effects were reported by 18 patients (52.9%), with hot flush and headache being the most frequently reported events. Previous hospitalization was reported by 13 patients (38.2%). Kinship information was valid for 100 of the 102 patient relatives. Psychological symptom scores according to kinship category were summarized descriptively. Inferential comparisons were not performed because some kinship categories contained very small numbers of participants, including only three participants in the uncle/aunt category.

BAI and BDI scores differed significantly among the three study groups (both *p* < 0.001; Table 3). Patients with IEI had a median BAI score of 19.0 (IQR: 12.75), patient relatives had a median score of 16.0 (IQR: 11.75), and healthy volunteers had a median score of 6.0 (IQR: 8.0). Dunn’s post hoc comparisons with Bonferroni adjustment showed no significant difference in BAI scores between patients and patient relatives (adjusted *p* = 1.000), whereas both patients and patient relatives had significantly higher BAI scores than healthy volunteers (both adjusted *p* < 0.001). Similarly, median BDI scores were 17.5 (IQR: 16.5) in patients, 13.0 (IQR: 10.75) in patient relatives, and 6.0 (IQR: 6.0) in healthy volunteers. BDI scores did not differ significantly between patients and patient relatives (adjusted *p* = 0.336), whereas both groups had significantly higher BDI scores than healthy volunteers (both adjusted *p* < 0.001).

**Figure 2:**
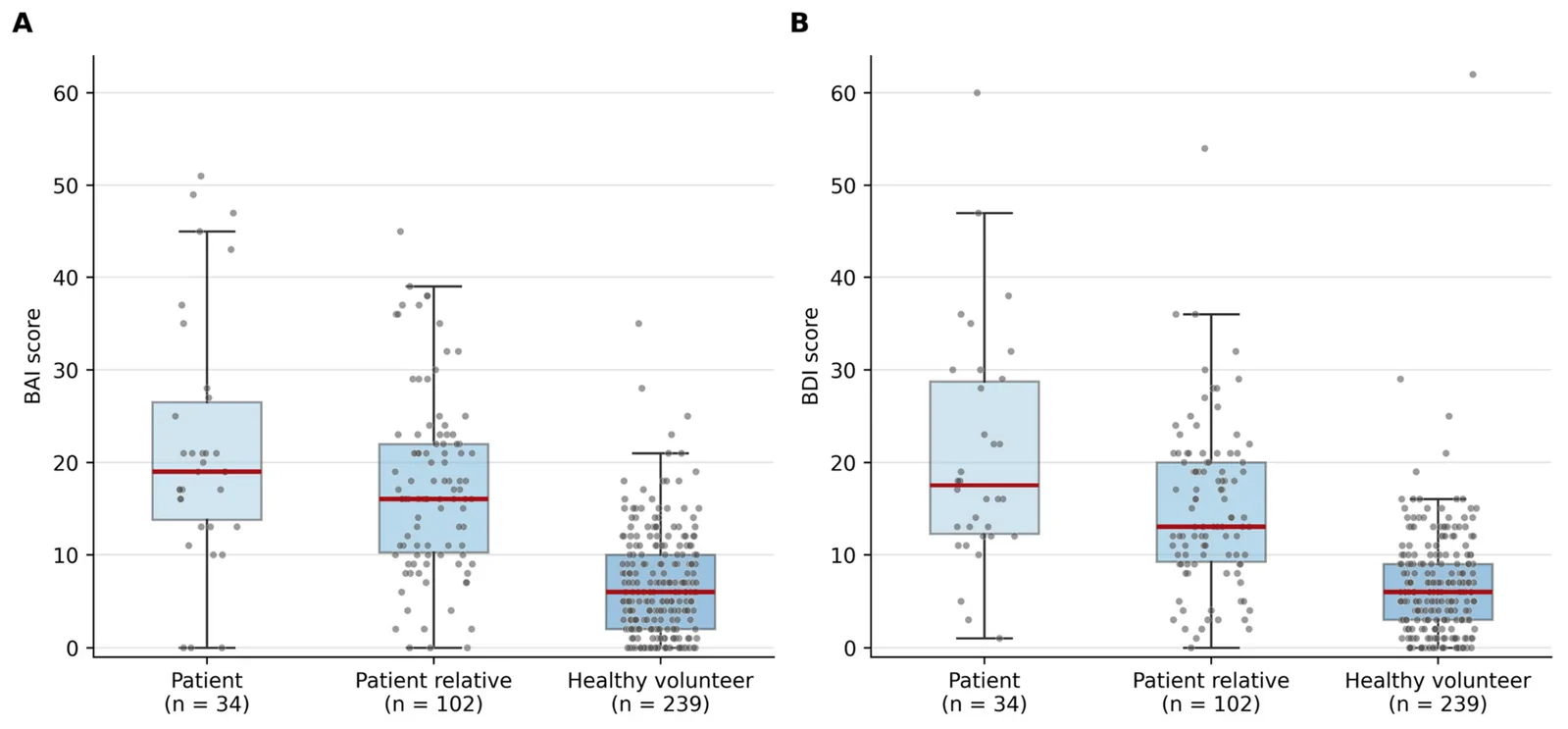
**Distribution of BAI and BDI scores across the study groups.** Individual observations are shown as jittered points. Boxes represent the interquartile range, horizontal red lines indicate the median, and whiskers extend to 1.5 times the interquartile range. (**A**) Beck Anxiety Inventory scores; (**B**) Beck Depression Inventory scores. BAI, Beck Anxiety Inventory; BDI, Beck Depression Inventory.

The distribution of Beck Anxiety and Beck Depression scores across groups is illustrated in Figure 2, visually supporting the statistical findings. Patients showed greater variability and higher peak values for both scales, whereas patient relatives demonstrated intermediate but heterogeneous distributions. In contrast, healthy volunteers exhibited lower and more narrowly distributed scores with minimal outliers.

Secondary exploratory comparisons of BAI and BDI scores according to sociodemographic characteristics are presented in Table 4. After Benjamini–Hochberg FDR adjustment, female participants had significantly higher BAI and BDI scores than male participants (FDR-adjusted *p* < 0.001 and *p* = 0.013, respectively). Participants with a chronic disease also had higher BAI and BDI scores than those without a chronic disease (FDR-adjusted *p* = 0.003 and *p* = 0.032, respectively). Unemployed participants demonstrated higher BAI and BDI scores than employed participants (FDR-adjusted *p* < 0.001 and *p* = 0.004, respectively). Monthly household income was significantly associated with both outcomes (FDR-adjusted *p* = 0.002 for BAI and *p* = 0.004 for BDI). Dunn–Bonferroni comparisons showed that participants in the highest-income category had significantly lower BAI and BDI scores than participants in both lower-income categories, whereas the two lower-income categories did not differ significantly from each other. Marital status was also associated with BAI and BDI scores after FDR adjustment; however, these results should be interpreted cautiously because the divorced and widowed categories contained only five and three participants, respectively. Educational level remained associated with BDI scores (FDR-adjusted *p* = 0.037) but not with BAI scores (FDR-adjusted *p* = 0.058). Age group was not associated with either outcome.

The results of the multivariable linear regression analyses with HC3 heteroscedasticity-consistent robust standard errors are presented in Table 5. To account for potential confounding arising from baseline differences in sociodemographic characteristics among groups, participant group, sex, age group, chronic disease status, employment status, monthly household income, and education level were simultaneously entered into the regression models.

In the BAI model, healthy volunteers had significantly lower anxiety scores than patients with IEI (B = −14.78, robust SE = 2.43, 95% CI: −19.55 to −10.00; *p* < 0.001). Although patient relatives also had lower BAI scores than patients, this difference was not statistically significant when HC3 robust standard errors were used (B = −4.14, robust SE = 2.52, 95% CI: −9.09 to 0.81; *p* = 0.101). Female sex was independently associated with higher BAI scores (B = 3.38, robust SE = 0.86, 95% CI: 1.68 to 5.08; *p* < 0.001). Age group, chronic disease status, employment status, household income, and educational level were not independently associated with BAI scores. Although the unadjusted Dunn post hoc analyses showed no statistically significant differences between patients and their relatives for either outcome, the adjusted analyses demonstrated significantly lower BDI scores among patient relatives, whereas the adjusted BAI difference remained statistically non-significant. These findings suggest that differences in measured sociodemographic characteristics may have influenced the unadjusted BDI comparison. However, given the small patient group and the exploratory nature of the multivariable analyses, these results should be interpreted cautiously. This finding indicates that baseline differences between the groups influenced the unadjusted comparisons and underscores the importance of adjusted analyses when interpreting between-group differences.

In the BDI model, both healthy volunteers and patient relatives had significantly lower depressive symptom scores than patients with IEI. The adjusted coefficient was −14.01 for healthy volunteers (robust SE = 2.35, 95% CI: −18.62 to −9.40; *p* < 0.001) and −5.69 for patient relatives (robust SE = 2.45, 95% CI: −10.51 to −0.86; *p* = 0.021). Female sex was not independently associated with BDI scores (B = 1.61, robust SE = 0.93, 95% CI: −0.22 to 3.44; *p* = 0.085). No independent associations were identified for age group, chronic disease status, employment status, household income, or educational level.

The BAI model explained 40.6% of the variance in anxiety scores (R^2^ = 0.406, adjusted R^2^ = 0.386; F(12, 362) = 20.63, *p* < 0.001), whereas the BDI model explained 31.6% of the variance in depressive symptom scores (R^2^ = 0.316, adjusted R^2^ = 0.293; F(12, 362) = 13.93, *p* < 0.001). The maximum variance inflation factor was 3.46, indicating no problematic multicollinearity. The Breusch–Pagan test indicated heteroscedasticity in the BAI model (*p* < 0.001) but not in the BDI model (*p* = 0.256). Cook’s distance values were below 1 in both models, although several observations showed elevated influence. HC3 robust standard errors were therefore retained to reduce sensitivity to heteroscedasticity and influential observations.

The internal consistency of both scales was evaluated using classical and modern reliability coefficients. For the BAI, Cronbach’s alpha (0.924), McDonald’s ω (0.923), and Guttman’s λ2 (0.926) indicated excellent internal consistency. Similarly, the BDI demonstrated excellent internal consistency, with Cronbach’s alpha of 0.939, McDonald’s ω of 0.940, and Guttman’s λ2 of 0.941.

The clinical and immunological classification of patients with inborn errors of immunity according to the 2024 International Union of Immunological Societies (IUIS) update is presented in Table 6. Among the 34 patients included in the study, the majority were classified as having predominantly antibody deficiencies (*n* = 28, 82.35%), with common variable immunodeficiency (CVID) representing the most frequent diagnosis (*n* = 22). Other antibody deficiencies included Bruton tyrosine kinase deficiency (X-linked agammaglobulinemia), activation-induced cytidine deaminase deficiency, and TWEAK deficiency. Combined immunodeficiencies affecting both T-cell and B-cell immunity were identified in two patients (5.88%), including RAG-related T−B− severe combined immunodeficiency and STK4 deficiency. Syndromic combined immunodeficiency was observed in one patient with autosomal dominant hyper-IgE syndrome due to STAT3 deficiency. Additional rare immune disorders included autoinflammatory disease caused by TREX1 deficiency (Aicardi–Goutières syndrome), complement deficiency due to C1q defects, and bone marrow failure syndrome related to Fanconi anemia (each *n* = 1, 2.94%). The mean age at diagnosis was 28.10 ± 21.28 years, with a mean follow-up duration of 10.51 ± 8.29 years. A positive family history of primary immunodeficiency was reported in 17.64% of patients, and a confirmed molecular genetic diagnosis was established in 35.29%.

## 4. Discussion

This study demonstrates that both patients with IEI and their family members exhibited significantly higher anxiety and depressive symptom scores than healthy controls. Although no significant differences were observed between patients and their relatives in the unadjusted analyses, multivariable regression analyses demonstrated modestly lower anxiety and depressive symptom scores among relatives after adjustment for potential sociodemographic confounders. The coexistence of elevated symptom scores in both groups suggests that the psychological context of IEI may extend beyond the affected individual and involve the broader family environment. Importantly, the difference between the unadjusted and adjusted findings indicates that sociodemographic and clinical characteristics may partly influence the apparent similarity between patients and their relatives. Thus, these analyses provide complementary rather than contradictory perspectives on group differences. In the adjusted multivariable models, participant group remained independently associated with both anxiety and depressive symptom scores, while female sex was independently associated with higher anxiety scores. Employment status, chronic disease status, income level, and education were not independently associated with psychological symptom scores after adjustment. The excellent internal consistency of the Beck Anxiety and Depression inventories indicates that both instruments demonstrated good reliability in the present study population.

Our results are consistent with accumulating evidence indicating that IEI imposes a considerable psychosocial burden on both patients and their families. Studies focusing on parents with IEI have similarly reported elevated anxiety and depression levels, emphasizing the need for psychosocial support to improve family well-being [7]. Broader syntheses describing the experience of “living with IEI” further identify anxiety and depression as prevalent and functionally limiting symptoms that impair social participation and family functioning, supporting the notion that the psychological impact of IEI is not confined to patients alone [8]. In line with our findings, Gumusburun et al. [12] reported high levels of anxiety and depressive symptoms accompanied by impaired psychosocial functioning in adults with IEI. Taken together, these observations suggest that psychological symptoms represent an important dimension of the overall experience of living with IEI rather than merely an accompanying clinical feature. Nevertheless, the cross-sectional nature of the present study does not allow determination of whether these symptoms are directly attributable to IEI or to other clinical and psychosocial factors.

The predominance of antibody deficiencies, particularly common variable immunodeficiency (CVID), should be considered when interpreting the psychological findings of the present cohort. Previous literature suggests that factors such as diagnostic delay, recurrent infections, chronic organ involvement, immune phenotype, and long-term treatment requirements may contribute to psychological symptoms in individuals with IEI. However, these disease-related characteristics were not specifically evaluated as predictors of anxiety or depressive symptoms in the present study. Similarly, caregiving intensity and disease-related care requirements were not directly assessed among family members. Therefore, any potential contribution of diagnostic delay, immune phenotype, disease complexity, or treatment requirements to the observed psychological symptoms remains hypothetical and should be investigated in future prospective studies.

A particularly notable finding of the present study is the absence of significant differences in anxiety and depressive symptom scores between patients and their relatives in the unadjusted analyses, suggesting a shared pattern of psychological symptoms within the family context. This observation aligns with literature indicating that family members involved in the care of individuals with chronic diseases may experience psychological symptoms related to uncertainty, repeated health-care utilization, and changes in family roles [13]. However, caregiving burden was not directly assessed in the present study. From a clinical perspective, the elevated symptom scores observed among relatives are noteworthy because they suggest that family members may themselves constitute a psychologically vulnerable group despite not being directly affected by the immune disorder. At the same time, the lower adjusted scores among relatives indicate that the magnitude of psychological symptoms may differ after accounting for sociodemographic and clinical characteristics. The present data cannot determine which specific family-, disease-, or care-related factors contribute to these symptoms, and these mechanisms warrant direct investigation in future studies.

In the unadjusted analyses, unemployment and lower household income were associated with higher psychological symptom scores; however, these associations were attenuated and no longer statistically significant after multivariable adjustment. In the adjusted models, female sex remained significantly associated with higher anxiety symptom scores but not with depressive symptom scores. Evidence indicates that depressive symptoms are more prevalent among female caregivers and may be related to social and health-related vulnerabilities [14]. The persistence of the association between female sex and anxiety after adjustment suggests that women may represent a subgroup with greater vulnerability to anxiety symptoms in this cohort. In contrast, the attenuation of the associations with income and employment after adjustment indicates that the socioeconomic differences observed in unadjusted analyses may partly reflect the distribution of other demographic or clinical characteristics rather than independent effects. Given the relatively small patient sample and exploratory nature of the multivariable analyses, these interpretations should be considered hypothesis-generating and require confirmation in larger cohorts.

A recent systematic review and meta-analysis demonstrated impaired health-related quality of life among individuals with IEI, including adverse effects across psychological and social domains [15]. IEI-specific studies have also demonstrated elevated anxiety and depressive symptoms and impaired quality of life among parents and caregivers of affected children [,], supporting the relevance of considering psychological well-being at the family level. However, these studies primarily focused on parents or caregivers of pediatric patients, whereas the present study extends the existing literature by simultaneously evaluating adults with IEI, their adult family members, and healthy controls within the same analytical framework. This distinction is clinically relevant because psychological experiences among adult relatives of adults with IEI may differ from those of parents caring for affected children, yet this population remains comparatively understudied.

The familial dimension of psychological symptoms is further supported by Kuburovic et al. [16], who reported impaired health-related quality of life and increased anxiety and depressive symptoms in children with IEI. Although pediatric findings cannot be directly extrapolated to the adult population examined here, they reinforce the broader concept that the psychosocial consequences associated with IEI may occur within a family context rather than exclusively at the level of the affected individual.

Emerging evidence suggests that immune dysregulation and chronic inflammation may contribute to psychiatric vulnerability in individuals with IEI [6,17,18]. Consistent with this possibility, Isung et al. [6] reported an increased risk of psychiatric disorders and suicidal behavior among individuals with primary humoral immunodeficiencies. However, such patient-specific biological mechanisms cannot directly explain the elevated anxiety and depressive symptom scores observed among family members in the present study. Psychological symptoms among relatives may instead be associated with psychosocial or family-related factors; however, these mechanisms were not directly assessed and therefore remain speculative. Heath et al. [19] reported that adults with IEI attributed a substantial proportion of their anxiety and depressive symptoms to their immunodeficiency, highlighting the potential role of illness perception in psychological distress. Similarly, Ahmed Meelad et al. [20] demonstrated impaired health-related quality of life among patients with IEI and their families, supporting the relevance of considering psychological well-being at the family level. Together, these findings suggest that biological and psychosocial pathways may contribute differently to psychological symptoms in patients and family members. The present study cannot distinguish these pathways but highlights the importance of evaluating patients and relatives as related yet clinically distinct groups. Whether systematic psychological screening or targeted psychosocial interventions improve psychological symptoms, treatment adherence, or quality of life requires evaluation in prospective studies.

Although lower household income was associated with higher anxiety and depression scores in univariable analyses, this association was attenuated after adjustment for participant group and other sociodemographic variables. Socioeconomic disadvantage may nevertheless be relevant to the psychological experience of families affected by chronic and rare diseases through factors such as treatment-related expenses, work disruption, and access to supportive resources, although these factors were not directly assessed in the present study. Similar associations between socioeconomic disadvantage and poorer psychological outcomes have been reported in rare disease populations [21,22]. Accordingly, the income-related differences observed in our unadjusted analyses should be interpreted cautiously and should not be considered evidence of an independent association with psychological symptoms. Further studies specifically examining socioeconomic and care-related factors in IEI families are warranted.

While previous studies have documented psychological symptoms or impaired quality of life in patients with IEI and, in some cases, their caregivers or families [9,16,19,20,23], the present study extends this literature by simultaneously evaluating adults with IEI, their adult family members, and healthy controls within the same analytical framework. This design allowed direct comparison of anxiety and depressive symptom scores across the three groups and assessment of whether the observed group differences persisted after adjustment for relevant sociodemographic factors.

### 4.1. Practical Implications

The present findings suggest that psychological well-being may warrant consideration not only in individuals with IEI but also among their family members. The elevated anxiety and depressive symptom scores observed in both groups indicate that a family-centered perspective may be relevant when planning comprehensive care. From a practical perspective, these findings may inform the future development of multimodal rehabilitation programs that integrate medical management with psychological assessment, psychoeducation, and individualized psychosocial support. Such programs could involve coordinated input from clinical immunologists, mental health professionals, rehabilitation specialists, and other healthcare professionals according to individual needs.

Importantly, the comparable elevation of psychological symptom scores among patients and relatives in the unadjusted analyses suggests that future rehabilitation models may benefit from considering family members as potential participants in the care process rather than focusing exclusively on the patient. Depending on identified needs, a multimodal approach could incorporate psychological symptom assessment, education regarding disease management, coping and stress-management strategies, and referral for appropriate psychological or psychiatric support. The association between female sex and higher anxiety scores may also help identify a subgroup in whom closer attention to anxiety symptoms could be considered, although this finding requires confirmation in larger cohorts.

Nevertheless, the present study did not evaluate the effectiveness of any rehabilitation, psychological, or psychosocial intervention. Therefore, these findings should not be interpreted as evidence that a specific multimodal rehabilitation program improves anxiety, depression, treatment adherence, or quality of life. Prospective intervention studies are needed to determine which components are effective, which patients and family members are most likely to benefit, and whether family-centered multimodal rehabilitation results in clinically meaningful improvements.

### 4.2. Advantages and Disadvantages of the Study

This study has several notable advantages. To our knowledge, it is among the few investigations to simultaneously evaluate anxiety and depressive symptoms in adults with IEI, their adult family members, and healthy controls within a single analytical framework. The simultaneous assessment of these three groups provides an opportunity to examine psychological symptoms from both patient- and family-level perspectives. In addition, the use of validated psychometric instruments with excellent internal consistency enhances the reliability of symptom assessment. Multivariable analyses provided complementary adjusted estimates; however, given the relatively small patient sample and baseline differences among groups, these analyses should be regarded as exploratory.

However, several limitations should be acknowledged. The cross-sectional design precludes causal inferences regarding the observed associations. Psychological symptoms were assessed using self-report questionnaires rather than structured clinical interviews; therefore, the findings reflect symptom severity rather than clinically confirmed psychiatric diagnoses. Participants receiving ongoing psychiatric treatment were excluded, which may have resulted in underestimation of psychological symptom severity; moreover, previous psychiatric diagnoses, psychotropic medication use, and prior psychological treatment were not systematically recorded. Although relatives’ relationship to the patient was documented, caregiving intensity, cohabitation, duration of caregiving, and time devoted to care were not assessed; therefore, the findings should not be interpreted as a direct measure of caregiver burden. Because the anonymous survey did not include family identifiers, relatives could not be linked to individual patients or to other participating relatives from the same family. Consequently, potential within-family clustering and the resulting non-independence of observations among relatives could not be assessed or accounted for statistically. This unaccounted within-family correlation may have affected the precision of the estimated standard errors and confidence intervals; therefore, findings concerning the relative group should be interpreted with particular caution. Future studies should record family identifiers and use statistical approaches that explicitly account for within-family clustering. The number of individuals approached and response rates were not systematically recorded. In addition, healthy controls were recruited through voluntary community participation and were not individually or frequency-matched to the patient or relative groups, and information on whether they were healthcare personnel or relatives of healthcare personnel was not systematically collected. Therefore, participation and selection bias cannot be excluded. The study groups also differed significantly in several sociodemographic and clinical characteristics, including income, employment, education, and chronic disease prevalence. Given the relatively small patient group, unequal group sizes, and sparse observations in some covariate categories, the multivariable regression estimates may have limited precision and coefficient stability despite the use of HC3 robust standard errors. Accordingly, the adjusted estimates should be considered exploratory and interpreted cautiously, and residual confounding cannot be excluded. The IEI cohort was clinically heterogeneous, comprising disorders with distinct clinical and immunological characteristics. Given the small and uneven numbers across diagnostic categories, subtype-specific analyses were not feasible. Accordingly, the findings should be interpreted as reflecting psychological symptoms in the overall study cohort, particularly among patients with predominantly antibody deficiencies, and should not be extrapolated to individual IEI subtypes. No formal *a priori* sample size calculation was performed because all eligible patients identified during the study period were invited to participate. Finally, the single-center design may further limit the generalizability of the findings. Despite these limitations, the findings indicate elevated anxiety and depressive symptom levels among both patients with IEI and their family members and support further investigation of family-level psychological well-being in larger, prospective studies.

## 5. Conclusions

In conclusion, adults with IEI and their family members exhibited significantly higher anxiety and depressive symptom scores than healthy controls. Although patients and relatives showed comparable symptom scores in unadjusted comparisons, multivariable analyses demonstrated modestly lower anxiety and depressive symptom scores among relatives after adjustment for sociodemographic and clinical factors. Participant group remained significantly associated with both anxiety and depressive symptom scores, while female sex was additionally associated with higher anxiety scores. These findings indicate that elevated psychological symptoms are observed not only among adults with IEI but also among their family members and support consideration of psychological well-being from a family-centered perspective. Given the cross-sectional design and exploratory nature of the adjusted analyses, larger prospective studies are needed to confirm these associations and evaluate the potential value of family-centered psychological and supportive interventions.

## Figures and Tables

**Table 1:** Sociodemographic characteristics of the study groups.

Variable	Patient (*n* = 34)	Patient Relative (*n* = 102)	Healthy Volunteer (*n* = 239)	*p* Value
BMI (kg/m^2^), mean ± SD	23.54 ± 5.30	26.71 ± 5.06	25.68 ± 4.23	0.005 ^a^
BMI (kg/m^2^), median (IQR)	23.55 (6.91)	25.69 (6.72)	25.88 (6.50)	
**Sex, ** ***n *** **(%)**				**0.535 ^b^**
Female	14 (41.2)	53 (52.0)	121 (50.6)	
Male	20 (58.8)	49 (48.0)	118 (49.4)	
**Age group, ** ***n *** **(%)**				**0.148 ^c^**
18–30 years	15 (44.1)	37 (36.3)	93 (38.9)	
30–65 years	19 (55.9)	55 (53.9)	137 (57.3)	
>65 years	0 (0.0)	10 (9.8)	9 (3.8)	
**Marital status, ** ***n *** **(%)**				**0.090 ^c^**
Married	16 (47.1)	67 (65.7)	142 (59.4)	
Divorced	0 (0.0)	3 (2.9)	2 (0.8)	
Widowed	1 (2.9)	1 (1.0)	1 (0.4)	
Single	17 (50.0)	31 (30.4)	94 (39.3)	
**Smoking, ** ***n *** **(%)**				**0.070 ^b^**
Yes	9 (26.5)	37 (36.3)	107 (44.8)	
No	25 (73.5)	65 (63.7)	132 (55.2)	
**Chronic disease, ** ***n *** **(%)**				**<0.001 ^b^**
Yes	26 (76.5)	35 (34.3)	62 (25.9)	
No	8 (23.5)	67 (65.7)	177 (74.1)	
**Employment status, ** ***n *** **(%)**				**0.003 ^b^**
Employed	15 (44.1)	52 (51.0)	160 (66.9)	
Unemployed	19 (55.9)	50 (49.0)	79 (33.1)	
**Monthly household income, ** ***n*** **(%)**				**<0.001 ^b^**
<27,000 TL	10 (29.4)	31 (30.4)	29 (12.1)	
27,000–88,000 TL	22 (64.7)	58 (56.9)	138 (57.7)	
>88,000 TL	2 (5.9)	13 (12.7)	72 (30.1)	
**Educational level, ** ***n *** **(%)**				**<0.001 ^c^**
Illiterate	1 (2.9)	3 (2.9)	0 (0.0)	
Primary school	10 (29.4)	25 (24.5)	24 (10.0)	
High school	9 (26.5)	31 (30.4)	64 (26.8)	
University	14 (41.2)	43 (42.2)	151 (63.2)	

**Notes:** Data are presented as *n* (%) unless otherwise indicated. BMI data were available for 34 patients, 101 patient relatives, and 237 healthy volunteers; three physically implausible height/weight records were excluded only from BMI calculations. Percentages for categorical variables were calculated using the full group denominators. SD, standard deviation; IQR, interquartile range; BMI, body mass index; TL, Turkish lira. **Tests:** ^a^ Kruskal–Wallis test; ^b^ Pearson chi-square test; ^c^ Fisher–Freeman–Halton exact test. All *p* values are two-sided.

**Table 2:** Clinical and treatment-related characteristics of patients with inborn errors of immunity and kinship characteristics of participating relatives.

Characteristic	*n* (%)
**Patients with IEI (** ***n*** ** = 34)**	
**Age at diagnosis**	
<6 years	6 (17.6)
6–18 years	7 (20.6)
18–40 years	9 (26.5)
>40 years	12 (35.3)
**Family history of IEI**	
Yes	14 (41.2)
No	20 (58.8)
**Route of immunoglobulin replacement**	
Intravenous	31 (91.2)
Subcutaneous	2 (5.9)
Facilitated subcutaneous	1 (2.9)
**Medication-related side effect**	
Yes	18 (52.9)
No	16 (47.1)
**Reported side effects among patients with side effects (** ***n*** ** = 18) ^a^**	
Hot flush	11 (61.1)
Headache	8 (44.4)
Fever	5 (27.8)
Rash	3 (16.7)
Palpitation	3 (16.7)
Dyspnea	3 (16.7)
Generalized swelling	1 (5.6)
Diarrhea	1 (5.6)
**Previous hospitalization**	
Yes	13 (38.2)
No	21 (61.8)
**Patient relatives with valid kinship data (** ***n*** ** = 100) ^b^**	
Parent	35 (35.0)
Sibling	37 (37.0)
Uncle/Aunt	3 (3.0)
Spouse	12 (12.0)
Child	13 (13.0)

**Notes:** Data are presented as *n* (%). IEI, inborn errors of immunity. **^a^** Patients could report more than one medication-related side effect; therefore, percentages for individual side effects do not sum to 100%. Percentages were calculated using patients who reported at least one side effect (*n* = 18). **^b^** Kinship information was unavailable or inconsistent for two of the 102 patient relatives. Percentages were calculated using the valid denominator (*n* = 100). The two relatives remained in all analyses not involving kinship.

**Table 3:** Comparison of Beck Anxiety Inventory and Beck Depression Inventory scores among the study groups.

Group	BAI, Mean ± SD	BAI, Median (IQR)	BDI, Mean ± SD	BDI, Median (IQR)
Patient (*n* = 34)	21.82 ± 13.32	19.0 (12.75)	20.59 ± 12.57	17.5 (16.5)
Patient relative (*n* = 102)	17.52 ± 9.47	16.0 (11.75)	15.13 ± 8.69	13.0 (10.75)
Healthy volunteer (*n* = 239)	6.78 ± 5.65	6.0 (8.0)	6.61 ± 6.09	6.0 (6.0)
Overall *p* value ^a^	<0.001		<0.001	
Patient vs. patient relative ^b^	1.000		0.336	
Patient vs. healthy volunteer ^b^	<0.001		<0.001	
Patient relative vs. healthy volunteer ^b^	<0.001		<0.001	

Data are presented as mean ± standard deviation and median (interquartile range). ^a^ Kruskal–Wallis test. ^b^ Dunn’s post hoc test with Bonferroni adjustment. Reported pairwise *p* values are multiplicity-adjusted *p* values. BAI, Beck Anxiety Inventory; BDI, Beck Depression Inventory; SD, standard deviation; IQR, interquartile range. All tests were two-sided.

**Table 4:** Beck Anxiety Inventory and Beck Depression Inventory scores according to sociodemographic characteristics.

Characteristic	*n*	BAI Mean ± SD	BAI Median (IQR)	BAI Unadjusted *p*	BAI FDR-Adjusted *p*	BDI Mean ± SD	BDI Median (IQR)	BDI Unadjusted *p*	BDI FDR-Adjusted *p*
**Sex**				**<0.001 ^a^**	**<0.001**			**0.005 ^a^**	**0.013**
Female	188	12.84 ± 9.77	11.0 (11.0)			11.05 ± 8.61	10.0 (10.0)		
Male	187	9.28 ± 9.28	7.0 (10.0)			9.34 ± 9.49	7.0 (9.0)		
**Age group**				**0.863 ^b^**	**0.863**			**0.936 ^b^**	**0.936**
18–30 years	145	11.23 ± 9.85	8.0 (12.0)			10.48 ± 9.36	8.0 (8.0)		
30–65 years	211	10.84 ± 9.50	9.0 (13.0)			9.98 ± 8.95	8.0 (10.0)		
>65 years	19	12.21 ± 10.85	9.0 (13.5)			10.37 ± 8.84	7.0 (12.5)		
**Marital status**				**0.030 ^b^**	**0.042**			**0.010 ^b^**	**0.018**
Single	142	10.75 ± 10.51	7.0 (12.0)			9.96 ± 9.28	7.0 (8.0)		
Divorced	5	18.60 ± 7.54	16.0 (1.0)			19.00 ± 7.58	16.0 (4.0)		
Married	225	11.00 ± 9.15	9.0 (12.0)			10.00 ± 8.87	8.0 (9.0)		
Widowed	3	18.33 ± 4.62	21.0 (4.0)			20.67 ± 8.50	21.0 (8.5)		
**Chronic disease**				**0.001 ^a^**	**0.003**			**0.023 ^a^**	**0.032**
Yes	123	13.75 ± 11.50	12.0 (14.0)			11.79 ± 9.93	10.0 (11.0)		
No	252	9.75 ± 8.38	8.0 (10.0)			9.42 ± 8.56	7.0 (9.25)		
**Employment status**				**<0.001 ^a^**	**<0.001**			**0.001 ^a^**	**0.004**
Employed	227	9.30 ± 8.05	7.0 (11.0)			8.84 ± 7.84	7.0 (10.0)		
Unemployed	148	13.76 ± 11.27	11.0 (13.5)			12.28 ± 10.41	10.0 (12.25)		
**Monthly household income**				**0.001 ^b^**	**0.002**			**0.001 ^b^**	**0.004**
<27,000 TL	70	13.73 ± 10.70	11.0 (13.0)			13.26 ± 11.66	10.0 (13.75)		
27,000–88,000 TL	218	11.48 ± 10.06	9.0 (12.0)			10.20 ± 8.24	9.0 (8.75)		
>88,000 TL	87	7.87 ± 6.66	6.0 (7.5)			7.72 ± 8.05	6.0 (7.5)		
**Educational level**				**0.050 ^b^**	**0.058**			**0.031 ^b^**	**0.037**
Illiterate	4	18.25 ± 5.50	21.0 (2.75)			16.50 ± 11.39	17.0 (14.0)		
Primary school	59	13.58 ± 11.54	11.0 (15.0)			12.58 ± 9.11	10.0 (14.5)		
High school	104	11.35 ± 10.26	9.0 (12.0)			10.58 ± 9.73	9.0 (9.0)		
University	208	10.07 ± 8.69	8.0 (10.25)			9.21 ± 8.58	7.0 (9.0)		

**Notes:** Data are presented as mean ± standard deviation and median (interquartile range). Unadjusted *p* values were obtained from the Mann–Whitney U test for two-category variables and the Kruskal–Wallis test for variables with three or more categories. FDR-adjusted *p* values were calculated using the Benjamini–Hochberg procedure separately for the seven sociodemographic comparisons conducted for each outcome. BAI, Beck Anxiety Inventory; BDI, Beck Depression Inventory; SD, standard deviation; IQR, interquartile range; FDR, false discovery rate; TL, Turkish lira. All tests were two-sided. **Tests:** ^a^ Mann–Whitney U test; ^b^ Kruskal–Wallis test. Marital-status findings should be interpreted cautiously because the divorced and widowed categories included only five and three participants, respectively. **Pairwise comparisons:** For monthly household income, Dunn–Bonferroni adjusted *p* values for BAI/BDI were 0.301/0.259 for <27,000 versus 27,000–88,000 TL, <0.001/<0.001 for <27,000 versus >88,000 TL, and 0.016/0.020 for 27,000–88,000 versus >88,000 TL. Pairwise comparisons were not emphasized for marital status or educational level because of sparse categories and the exploratory nature of these analyses.

**Table 5:** Multivariable linear regression analyses of factors associated with Beck Anxiety Inventory and Beck Depression Inventory scores.

Variable	BAI B (Robust SE)	BAI 95% CI	BAI *p*	BDI B (Robust SE)	BDI 95% CI	BDI *p*
**Participant group**						
Patient relative vs. patient	−4.14 (2.52)	−9.09 to 0.81	0.101	−5.69 (2.45)	−10.51 to −0.86	0.021
Healthy volunteer vs. patient	−14.78 (2.43)	−19.55 to −10.00	<0.001	−14.01 (2.35)	−18.62 to −9.40	<0.001
**Sex**						
Female vs. male	3.38 (0.86)	1.68 to 5.08	<0.001	1.61 (0.93)	−0.22 to 3.44	0.085
**Age group**						
30–65 vs. 18–30 years	0.32 (0.94)	−1.53 to 2.16	0.738	0.07 (0.91)	−1.72 to 1.86	0.939
>65 vs. 18–30 years	0.36 (2.31)	−4.19 to 4.91	0.876	−0.61 (1.81)	−4.17 to 2.94	0.735
**Chronic disease**						
No vs. yes	−1.14 (0.87)	−2.85 to 0.57	0.192	0.37 (0.92)	−1.44 to 2.17	0.691
**Employment status**						
Unemployed vs. employed	1.46 (0.98)	−0.46 to 3.38	0.137	1.11 (1.01)	−0.87 to 3.08	0.272
**Monthly household income**						
25,000–82,000 vs. <25,000 TL	0.51 (1.37)	−2.18 to 3.20	0.708	−0.75 (1.34)	−3.39 to 1.89	0.578
>82,000 vs. <25,000 TL	0.09 (1.49)	−2.83 to 3.01	0.953	−0.74 (1.48)	−3.65 to 2.18	0.620
**Educational level**						
High school vs. primary school	1.18 (1.42)	−1.61 to 3.97	0.406	0.57 (1.34)	−2.07 to 3.22	0.670
Illiterate vs. primary school	−1.11 (4.48)	−9.92 to 7.69	0.804	−0.52 (7.57)	−15.42 to 14.38	0.945
University vs. primary school	1.27 (1.40)	−1.47 to 4.01	0.364	0.38 (1.28)	−2.14 to 2.89	0.768

**Notes:** Unstandardized regression coefficients are presented with HC3 heteroscedasticity-consistent robust standard errors, 95% confidence intervals, and two-sided *p* values. All variables shown were entered simultaneously. Reference categories were patient group, male sex, age 18–30 years, chronic disease present, employed status, monthly household income <27,000 TL, and primary-school education. BAI, Beck Anxiety Inventory; BDI, Beck Depression Inventory; B, unstandardized regression coefficient; SE, standard error; CI, confidence interval; TL, Turkish lira. **Model fit:** BAI model: R^2^ = 0.406, adjusted R^2^ = 0.386, F(12,362) = 20.63, *p* < 0.001. BDI model: R^2^ = 0.316, adjusted R^2^ = 0.293, F(12,362) = 13.93, *p* < 0.001. The maximum variance inflation factor was 3.46.

**Table 6:** Clinical and immunological classification of patients with inborn errors of immunity according to the 2024 IUIS update.

IUIS Category (2024)	Specific Diagnosis	*N* (%)
**Immunodeficiencies affecting both T-cell and B-cell immunity**	T–B– SCID (RAG deficiency)	1 (2.94)
	STK4 deficiency	1 (2.94)
**Combined immunodeficiencies with associated or syndromic features**	AD-HIES (STAT3 deficiency, Job syndrome)	1 (2.94)
**Predominantly antibody deficiencies**	BTK deficiency (X-linked agammaglobulinemia)	4 (11.76)
	AID deficiency	1 (2.94)
	TWEAK deficiency	1 (2.94)
	Common variable immune deficiency (CVID)	22 (64.71)
**Diseases of immune dysregulation**	–	–
**Congenital defects of phagocyte number or function**	–	–
**Defects in intrinsic and innate immunity**	–	–
**Autoinflammatory diseases**	TREX1 deficiency (Aicardi–Goutières syndrome type 1)	1 (2.94)
**Complement deficiencies**	C1q deficiency	1 (2.94)
**Bone marrow failure syndromes**	Fanconi anemia	1 (2.94)
**Phenocopies of IEI**	–	–
**Total**		**34 (100)**
**Additional clinical characteristics**		
**Variable**	**Value**	
***Age at diagnosis, years (mean ± SD)***	28.10 ± 21.28	
***Follow-up duration, years (mean ± SD)***	10.51 ± 8.29	
***Positive family history of IEI, n (%)***	6 (17.64)	
***Confirmed genetic diagnosis, n (%)***	12 (35.29)	

Classification was performed according to the 2024 International Union of Immunological Societies (IUIS) Expert Committee update on inborn errors of immunity. Percentages are calculated based on the total number of patients (*N* = 34). AD-HIES: autosomal dominant hyper-IgE syndrome; AID: activation-induced cytidine deaminase; BTK: Bruton tyrosine kinase; CVID: common variable immune deficiency; IEI: inborn errors of immunity; SCID: severe combined immunodeficiency. Dashes (–) indicate that no patients in the cohort were classified under the corresponding category.

## Data Availability

The data that supports the findings of this study are available on request from the corresponding author. The data is not publicly available due to privacy or ethical restrictions.
